# Dual Roles of Reducing Systems in Protein Persulfidation and Depersulfidation

**DOI:** 10.3390/antiox14010101

**Published:** 2025-01-16

**Authors:** Zhichao Liu, Nicolas Rouhier, Jérémy Couturier

**Affiliations:** 1 Université de Lorraine, INRAE, IAM, F-54000 Nancy, France; 2 Institut Universitaire de France, F-75000 Paris, France

**Keywords:** hydrogen sulfide, protein persulfidation, thioredoxin, glutaredoxin, glutathione, sulfurtransferase

## Abstract

The oxidative modification of specific cysteine residues to persulfides is thought to be the main way by which hydrogen sulfide (H_2_S) exerts its biological and signaling functions. Therefore, protein persulfidation represents an important thiol-switching mechanism as other reversible redox post-translational modifications. Considering their reductase activity but also their connections with proteins that generate H_2_S and its related molecules, the glutaredoxin (GRX) and thioredoxin (TRX)-reducing systems have potential dual roles in both protein persulfidation and depersulfidation. In this review, we will first focus on recent advances describing the physiological pathways leading to protein persulfidation before discussing the dual roles of the physiological TRX and glutathione/GRX-reducing systems in protein persulfidation/depersulfidation.

## 1. Introduction

Protein persulfidation corresponds to the addition of an extra sulfur atom on a cysteine thiol group (-SH), leading to the formation of a persulfide group (-SSH) [1]. It represents a catalytic intermediate in cysteine desulfurases (CDs), sulfur carrier proteins and sulfurtransferases (STRs), proteins involved in the biosynthetic pathways of sulfur-containing cofactors (iron-sulfur (Fe-S) and molybdenum cofactors), or molecules (biotin, lipoic acid, sulfur-containing RNA bases) in both prokaryotic and eukaryotic systems [1,2]. Protein persulfidation has gained increasing attention over the last 15 years since it was proposed to represent the main way by which H_2_S exerts its biological functions [3]. This redox post-translational modification (PTM) is also suggested to prevent overoxidation of protein cysteine residues and the associated deleterious effects on protein function [4]. For instance, protein persulfidation increases in mouse embryonic fibroblasts and human HeLa cells after treatment with H_2_O_2_. However, in the oxidative environment of aging cells, total protein persulfidation was decreased while protein overoxidation (sulfinylation and sulfonylation) increased [4,5]. The protein persulfidation level was found to decrease with brain aging in rat and human neurodegeneration diseases such as Alzheimer’s disease [4,6], while an increase in protein persulfidation level extended the lifespan of *Caenorhabditis elegans* [4]. In the human pathogen *Aspergillus fumigatus*, a lower persulfidation level correlates with a higher susceptibility to host-mediated killing [7]. The authors also highlighted that the host persulfidation level determined the fungal persulfidation level and, consequently, its virulence. In the nosocomial bacterial pathogen *Staphylococcus aureus,* protein persulfidation has also been associated with virulence [8]. In addition to host–pathogen interactions, protein persulfidation is also modulated during the establishment of symbiosis. In the symbiotic nitrogen fixation model between common bean (*Phaseolus vulgaris*) and *Rhizobium leguminosarum*, a decrease in persulfidated proteins was observed for plant proteins during nodule aging [9]. Together with the decrease in antioxidants and the increase in oxidized proteins previously observed during nodule aging, these results point again to the role of protein persulfidation during aging, as described in animals [4]. A very high number of persulfidated proteins has been identified in leaves (2015 proteins) and roots (5214 proteins) from the model plant *Arabidopsis thaliana* (Arabidopsis) grown under normal conditions [10,11]. As observed for other organisms, protein persulfidation increases under stress conditions such as drought [12,13] and high CO_2_ (0.7% CO_2_ *v*/*v*) conditions that promote oxidative stress [14]. Therefore, protein persulfidation represents an important thiol-switching mechanism in all organisms as other redox PTMs, such as glutathionylation or nitrosylation [15]. In recent years, numerous studies have focused on unraveling the molecular mechanisms and protein players that control protein persulfidation and depersulfidation [16]. In this review, we focus on recent advances describing the physiological pathways leading to protein persulfidation and the dual roles of the thioredoxin (TRX) and glutathione/glutaredoxin (GRX) physiological reducing systems in both reactions.

## 2. Mechanisms of Protein Persulfidation in Cells

Over the last 15 years, protein persulfidation has been mainly associated with H_2_S signaling/metabolism. H_2_S production relies on multiple and specific pathways depending on the organisms and/or tissues/cells considered. In mammals and plants, these pathways have been described in previous reviews [17,18,19]. In an aqueous solution, there is a mixture between H_2_S and HS^−^, whose equilibrium depends on the pH and the temperature [20]. For instance, at a physiological pH of 7.4 and at 37 °C, the proportions of HS^−^ and H_2_S are 81 and 19%, respectively [20]. Moreover, from a thermodynamic point of view, H_2_S cannot react directly with a thiol group [20]. While the biochemical and cellular processes linking H_2_S to protein persulfidation are not yet entirely elucidated, several H_2_S-mediated protein persulfidation pathways have been proposed. Other more or less complex and efficient biochemical processes may exist. These pathways and processes are presented below.

### 2.1. Switch of Redox Post-Translational Modifications by H_2_S

**Figure 1:**
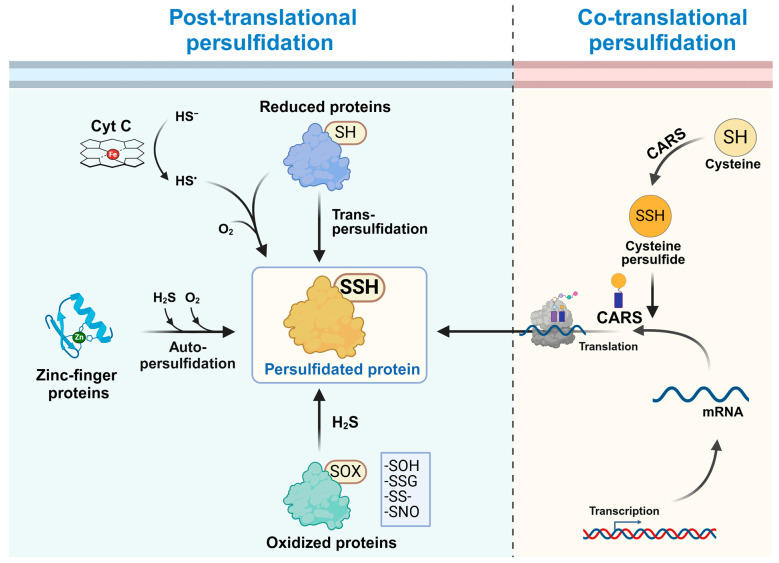
Molecular mechanisms promoting protein persulfidation in cells. Protein persulfidation is achieved by either post-translational or co-translational mechanisms and can possibly occur on either reduced or oxidized cysteine forms. Concerning post-translational pathways, it is possible to distinguish enzyme-based from non-enzyme-based mechanisms. The latter mechanisms rely on the reaction of H_2_S with oxidized cysteines present in proteins, either engaged in disulfide bond (S-S), sulfenylated (SOH), glutathionylated (SSG), or even nitrosylated (SNO), as postulated but not yet experimentally proven. In enzyme-based mechanisms, three situations have been distinguished so far. The first case is transpersulfidation reactions between reduced proteins and specific persulfidases/sulfurtransferases (STR or 3-mercaptopyruvate sulfurtransferase (MST)) under their persulfide state. The second and third cases are metal-catalyzed protein persulfidation mechanisms that occur in the presence of H_2_S and O_2_: one is the auto-persulfidation of Zn-finger proteins, the other is the heme-dependent formation of an HS• radical, which further reacts with protein thiols, as shown for cytochrome *c*. Finally, protein persulfidation can be co-translational when cysteinyl-tRNA synthetase (CARS) proteins incorporate cysteine persulfide, which they formed themselves, instead of cysteine onto tRNA. Created with BioRender.com.

As a reductant, H_2_S can potentially react with oxidized cysteines, involved for instance in a disulfide bond or being sulfenylated, nitrosylated, or glutathionylated (Figure 1). Several studies have focused on the H_2_S-dependent persulfidation of sulfenylated proteins, as shown in vitro for the bovine serum albumin (BSA), whose sulfenylated form is converted into a persulfidated form after reaction with H_2_S [21]. Kinetic analysis using sulfenylated human serum albumin (HSA) showed a reaction about 600 times faster with H_2_S than with reduced glutathione (GSH) [22]. A study performed with the sulfenylated form of the one-cysteine peroxiredoxin alkyl hydroperoxide reductase E from *Mycobacterium tuberculosis* revealed a reaction rate six times higher with H_2_S than with mycothiol [23]. These results indicate that the formation of sulfenic acids represents a very plausible intermediate for the H_2_S-dependent protein persulfidation and points to a possible intricate link between H_2_O_2_ and H_2_S-related or signaling pathways. Since H_2_O_2_ oxidizes specific cysteine residues of target proteins to the sulfenic acid form, the subsequent reaction with H_2_S would promote the specific persulfidation of target proteins. An increase in intracellular persulfide levels, which depends on the activity of H_2_S-producing enzymes, is observed in human neuroblastoma cells treated with H_2_O_2_ (which leads to an increase in both sulfenic acids and disulfides) [24]. On the contrary, human neuroblastoma cells treated with diamide, which promotes disulfide formation only, do not exhibit an increase in intracellular persulfides [24]. Therefore, the conversion of sulfenic acids into persulfides seems to represent a major reaction pathway for protein persulfidation [4,14].

It has been also demonstrated that H_2_S reacts with some low molecular weight (LMW) disulfides (cystine, homocystine, oxidized glutathione (GSSG)), as well as with the corresponding cysteinylated, homocysteinylated, and glutathionylated HSA [22]. These reactions yield the formation of cysteine persulfide (CysSSH), homocysteine persulfide, and glutathione persulfide (GSSH), respectively. However, the rate constants for the reactions with both LMW disulfides (ranging from 0.16 to 0.6 M^−1^ s^−1^) and HSA disulfides (0.23 to 3 M^−1^ s^−1^) are rather low [22]. Considering the low-rate constants for the H_2_S reduction of cysteinylated and glutathionylated HSA (3 and 1.7 M^−1^ s^−1^, respectively), and the low intracellular H_2_S concentration (estimated between 9 and 29 nM) [25], it is thought that disulfide reduction by H_2_S is unlikely to represent a major general reaction leading to protein persulfidation. Nevertheless, since the rate constants between disulfide and H_2_S are correlated with the thiol p*K_a_* of the leaving cysteine residue [22], which itself depends on the specific protein environment, it remains possible that the reduction by H_2_S of disulfide involving cysteines with acidic p*K_a_* leads to protein persulfidation, as suggested for two peroxiredoxin isoforms, Aspf3 and PrxA, from *A. fumigatus* [7]. Moreover, a redox switch from protein glutathionylation to protein persulfidation has been observed in human pancreatic cells under oxidative stress conditions [26]. Up to now, there is no direct evidence of a redox switch from protein S-nitrosylation to protein persulfidation despite computational studies pointing out that the reaction between nitrosothiol (SNO) and H_2_S would be possible for certain proteins possessing positively charged residues near the S-NO bond, which could stabilize the NO moiety of S-nitrosylated cysteine residues [27]. From the available evidence, it appears that the major protein persulfidation pathways mediated by H_2_S are preceded by the formation of sulfenic acids. The formation of disulfide bonds and/or glutathionylated cysteines may represent other minor pathways. Future work is expected to clarify these aspects.

### 2.2. Metal-Catalyzed Protein Persulfidation

Interactions of H_2_S with redox-active metal centers such as hemes or with non-redox-active metal centers such as those present in zinc finger-containing proteins are directly involved in protein persulfidation and in reactions that require the presence of oxygen (Figure 1) [20,28,29]. A role for heme iron atoms has been highlighted in vitro for cytochrome *c* (Cyt *c*), which catalyzes the one-electron oxidation of H_2_S into an HS• radical, which then reacts with protein thiols in the presence of O_2_ to form protein persulfides [28]. Accordingly, *Cyt c* silencing resulted in a small decrease in protein persulfidation in HeLa cells cultivated under normoxic conditions and diminished the increase in protein persulfidation observed in cells cultivated under hypoxic conditions [28]. In particular, Cyt *c* has been reported to stimulate the persulfidation of procaspase-9, a protein involved in apoptosis [28].

The first evidence for the role of Zn in H_2_S-dependent protein persulfidation was obtained using the Zn-finger protein tristetrapolin as a model and cryo-electrospray time-of-flight mass spectrometry [29]. The authors demonstrated that zinc facilitates the H_2_S-dependent protein persulfidation before the subsequent formation of disulfide only when O_2_ is present. Under the oxidized form, the protein loses its ability to bind to RNA while, when bound to RNA, the protein is protected from oxidation by H_2_S. More recently, the role of zinc has been analyzed in more details [30]. It is reported that a small molecule Zn^II^ coordination complex, mimicking Zn-finger binding sites, is able to coordinate HS^−^ and to catalyze the electron transfer from HS^−^ to O_2_ and the subsequent disulfide bond formation [30]. Numerous zinc finger proteins belonging to three common types (CCCC, CCCH, and CCHH) have been isolated as persulfidated proteins in a range of eukaryotes including, notably, mammalian and plant model organisms [31]. Hence, the H_2_S-dependent persulfidation of zinc finger proteins may represent a widespread auto-persulfidation mechanism.

### 2.3. Specific Protein Persulfidation Through Transpersulfidation Reactions

Due to the toxicity of free sulfide and the need for specific sulfur transfer reactions, stable non-toxic chemical species mediate the supply of sulfur in the biosynthetic pathways of various sulfur-containing compounds. Hence, in addition to examples where sulfur is provided by sacrificing Fe-S clusters, as in the case of biotin, thiamine, and lipoic acid synthesis [32], or by a GSH molecule, as in the case of camalexin synthesis [33], persulfide groups represent a major sulfur delivery source and an efficient sulfur relay system, in particular for the formation of Fe-S centers, molybdenum cofactors, and sulfur-containing bases in tRNA (thionucleosides) [34]. In these pathways, sulfur transfer reactions occur through the formation of persulfide groups on reactive cysteines. The transfer of a persulfide is referred to as a transpersulfidation reaction. In other words, one enzyme, that has extracted a sulfur atom from a donor substrate or received it from another persulfidated protein, transfers the persulfide to an acceptor protein/enzyme.

The protein families that catalyze transpersulfidation reactions play a more or less restricted role in the overall level of persulfidated proteins. For instance, DsrC and DsrEFH proteins are specifically responsible for cytoplasmic sulfur trafficking and oxidation in sulfur-oxidizing prokaryotes [35]. In some sulfur-oxidizing bacteria, sulfur trafficking also implicates two other protein families acting as sulfur carriers, Rhodanese (Rhd) and TusA [36]. While TusA-like proteins are specific to prokaryotes and provide sulfur for the synthesis of molybdenum cofactor or thionucleosides of tRNAs [37], Rhd-containing proteins, also named STRs, are widely distributed. Important differences among STR proteins in terms of primary sequences, protein domain architecture, and structure/length of the active site loop exist [1]. Most of them possess a conserved catalytic cysteine in the Rhd domain conferring the ability to catalyze transpersulfidation reactions, but their exact functions and partners are in many cases yet largely unknown [1,38,39,40,41]. Some STRs and TusA proteins interact with CDs. This implies that cysteine is the sulfur donor and connects the cysteine metabolism to many sulfur-trafficking pathways [2]. Indeed, CDs are responsible for the initial step of sulfur mobilization/extraction from cysteine. These are pyridoxal 5′-phosphate (PLP)-dependent enzymes that catalyze the desulfuration of cysteine to alanine, leading to the concomitant formation of a persulfide on their conserved catalytic cysteine. CDs are notably well known for their fundamental role as sulfur donors for Fe-S cluster biogenesis, molybdopterin synthesis, and sulfur insertion in thionucleosides. The subsequent direction of sulfur transfer is dictated by the interactions with various protein acceptors acting as sulfur carriers [42]. Recent bioinformatic analyses have highlighted the existence of natural CD-TusA and CD-Rhd fusion proteins for instance [43], supporting the experimental evidence that isolated proteins interact together and participate in successive transpersulfidation reactions. As for the first step, the direction of the sulfur flux is governed by the interactions with specific partners. While TusA function seems restricted to thionucleoside synthesis, some STR proteins might have a more general role in protein persulfidation. Such a role has been suggested for the Arabidopsis cytosolic sulfurtransferase 18 (STR18), which catalyzes the transpersulfidation reaction to roGFP2 in vitro, receiving the persulfide from the cytosolic CD isoform ABA3 [44], and for the human mitochondrial STR protein, Thiosulfate Sulfurtransferase-Like Domain-Containing 1 (TSTD1), which promotes the persulfidation of TRX [45]. Moreover, a proteomic analysis has recently identified 64 proteins whose persulfidation levels are decreased in human embryonic kidney 293 (HEK293) cells knockout for the 3-mercaptopyruvate sulfurtransferase (MST), a specific type of Rhd-containing protein [46]. Coupled with the ability to oxidize roGFP2, human MST has been proposed as a persulfidase [46]. Nevertheless, the relatively low number of proteins, whose persulfidation depends on MST, suggests a specific mechanism based on protein–protein interactions and not unspecific H_2_S-dependent mechanisms. Furthermore, a transpersulfidation reaction implies that the acceptor proteins are reduced while H_2_S-mediated persulfidation occurs on oxidized proteins (Figure 1).

LMW thiols can also act as acceptor molecules in transpersulfidation reactions that lead to the synthesis of LMW persulfides. For instance, in vitro experiments identified cysteine as both a substrate and a sulfur acceptor for the human CD isoform Nfs1, leading to the formation of CysSSH [47]. GSH is an acceptor for both Arabidopsis MST isoforms, STR1 and STR2 [48], and for the human sulfide quinone oxidoreductase [49]. These reactions generate GSSH. More recently, a specific CysSSH synthesis pathway was proposed to involve cysteinyl-tRNA synthetases (CARSs) [50]. In vitro, the *E. coli* PLP-binding CARS is able to convert Cys efficiently into CysSSH, a reaction that is dependent on PLP but independent of ATP and tRNA [50]. Hence, this cysteine persulfide synthase (CPERS) activity is independent of the function of aminoacyl-tRNA biosynthesis. Later on, these enzymes were proposed to be the principal enzymes responsible for the biosynthesis of reactive persulfides and polysulfides in mammalian cells [51]. Nevertheless, the physiological relevance of the direct protein persulfidation by LMW persulfides has been questioned due to unfavorable thermodynamic constraints for the tautomerization of LMW persulfide to a thiosulfoxide form, which is required for the transpersulfidation reaction to occur [52].

### 2.4. Co-Translational Protein Persulfidation

All mechanisms described above occur at the post-translational level. In addition to the synthesis of cysteine persulfide, CARS proteins have been also proposed to insert cysteine persulfide into proteins during translation [50] (Figure 1). Indeed, in a more classical tRNA aminoacylation reaction, it was shown that *E. coli* CARS is able to incorporate, not only Cys but also CysSSH, onto tRNA. Hence, CysSSH-bound tRNA is incorporated in ribosomes and CysSSH is incorporated into nascent polypeptides, as illustrated for GAPDH. These results illustrate the possibility that protein persulfidation may also be a co-translational process in addition to a post-translational modification. Mammals possess two CARS proteins, CARS1 and CARS2, localized in cytosol and mitochondria, respectively. Both human proteins display a strong CPERS activity in vitro but only CARS2 seems to play a predominant role in persulfide production in vivo. As observed for *E. coli* CARS, both CPERS and cysteinyl-tRNA synthetase activities of human CARS2 are independent [50]. The CRS1 ortholog from *Saccharomyces cerevisiae* is dual-targeted to the cytosol and mitochondria where it has different roles. The mitochondrial CRS1 contributes to the maintenance of mitochondrial energy metabolism while the cytosolic CRS1 participates in protein persulfidation [53]. Arabidopsis possesses three CARS proteins named SYCC1, SYCC2, and SYCO. Both SYCC1 and SYCC2 are predicted to be localized in the cytosol while SYCO is dual-targeted to chloroplasts and mitochondria [54]. Despite the CPERS activity of CARSs seems evolutionary conserved, whether plant isoforms participate in such a co-translational protein persulfidation process remains to be confirmed.

The overall importance and physiological significance of co-translational protein persulfidation remains unknown. Considering that oxidation of cysteine persulfides is reversible, introducing CysSSH during translation may be a useful mechanism to maintain protein synthesis under stress conditions. When conditions return to a normal state, physiological reductases should reduce the modifications and even possibly activate the proteins if persulfidation inhibits its function [55].

## 3. Dual Roles of Reducing Systems in Protein Persulfidation and Depersulfidation

### 3.1. Depersulfidation

**Figure 2:**
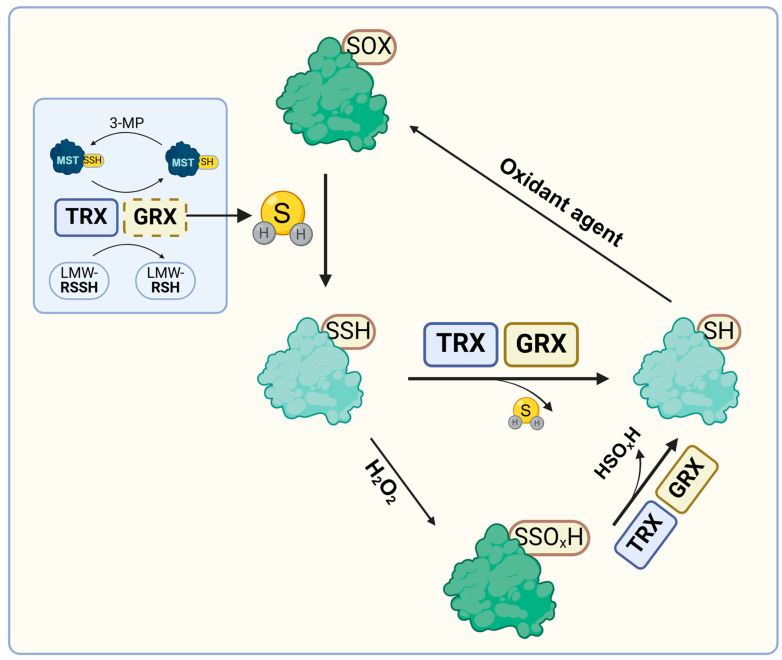
Dual roles of GRX and TRX systems in protein persulfidation/depersulfidation. On the one hand, some GRXs and TRXs are able to catalyze the reduction of persulfidated proteins (depersulfidation) or of oxidized persulfide groups (PSSO_X_H), such as perthiolsulfenylated (PSSOH), perthiolsulfinylated (PSSO_2_H), and perthiolsulfonylated forms (PSSO_3_H), which can be formed when the external sulfur atom of persulfides undergoes one or several consecutive oxidations mediated in particular by hydrogen peroxide. These reactions release H_2_S or HSO_x_H, respectively. On the other hand, the H_2_S pool either formed upon depersulfidation reactions or generated by the interaction of TRX and potentially GRX with sulfurtransferases, and more particularly MST isoforms, may promote non-enzymatic protein persulfidation upon reaction with sulfenylated proteins. Created with BioRender.com.

Similar to other redox PTMs, persulfidation is a reversible mechanism [15]. Depersulfidation reactions are catalyzed by the glutathione/glutaredoxin and thioredoxin reducing systems (Figure 2). For instance, TRX efficiently reduces persulfidated cysteines in the protein tyrosine phosphatase 1B (PTP1B) and in human and bovine serum albumins (HSA and BSA) but also free CysSSH in vitro [16,24,56]. Accordingly, cells knockdown for *TrxR1* or treated with auranofin, a TRX reductase (TrxR) inhibitor, display an increased total intracellular persulfidation level [16,24]. In a similar manner, the GSH/GRX system efficiently reduces persulfidated BSA in vitro [16]. Furthermore, in mouse hepatocytes lacking both TrxR and glutathione reductase (GR), the level of persulfidated proteins is markedly elevated [16]. Finally, the distinct patterns of persulfidated proteins in wild-type, *GR*-null and *TrxR*/*GR*-null cells suggest specific roles for both systems, which might be potentially involved in different signaling pathways [16,55].

Altogether, these data underline the importance of both reducing systems for protein depersulfidation despite their exact contribution and the potential ability of some TRXs/GRXs to act as specific depersulfidases remain unclear. For instance, among TRXs tested, the human TRX-related protein 14 (TRP14) efficiently reduces persulfidated forms of PTP1B, Prx2, HSA, and BSA [16,24]. However, only a moderate increase in persulfidated proteins was observed in the liver of *TRP14*-null mice compared to WT mice, suggesting that TRP14 participates only in the reduction of specific persulfidated proteins and that other depersulfidases exist [55]. In fact, while it does not reduce the targets of regular TRXs, human TRP14 also possesses S-denitrosylase activity in vitro, being as efficient as human Trx1, and cystine reductase activity, being in this case more efficient than human Trx1 [57]. Coupled with in vivo genetic studies performed in *C. elegans*, mice, and human, TRP14 has thus been recently proposed as an evolutionarily conserved enzyme, possibly primarily involved in cystine reduction and in the decysteinylation of proteins [58]. It is worth noting that TRP14 family members have a conserved non-canonical WCPDC signature that might contribute to these particular biochemical properties [59]. In fact, a TRX-like isoform from *S. aureus*, named TrxP and displaying the same WCPDC motif, has been proposed to act as a depersulfidase under conditions of sulfide stress [60]. In this organism, the canonical TrxA may also contribute significantly to depersulfidation, but rather under normal growth conditions. Regarding plants, TRP14 orthologs are poorly characterized, and a connection with depersulfidation has not yet been established. In fact, poplar Clot/TRP14 does not exhibit any significant reductase activity when tested using insulin or the chloroplastic NADP-malate dehydrogenase [61]. Hence, it appears that TRP14-type proteins exert rather specific biochemical functions, including depersulfidase activity, even though some of these functions are shared by multiple TRXs. Therefore, the subcellular localization and expression patterns may be important. As human TRP14, plant Clot/TRP14 is localized in the cytosol as demonstrated for the poplar protein and predicted for other plant orthologs [61]. This specific localization implies that other proteins with depersulfidase activity should be present in other subcellular compartments, notably in mitochondria and chloroplasts considering that numerous proteins from these compartments have been identified as persulfidated [10,11,12,14]. While the set of mitochondrial TRXs/GRXs in photosynthetic eukaryotes is comparable to the one in other eukaryotes, photosynthetic eukaryotes possess a large set of plastidial GRXs and TRXs, including many atypical TRXs, the activity profile of which is not completely delineated [62,63]. In addition to the systematic in vitro assays using model protein substrates, studying the role of specific TRX/GRX proteins will require comprehensive proteomic studies performed on knock-out or overexpressing cells/organisms to identify the specificity towards the reduction of persulfidated proteins.

### 3.2. Reduction of Oxidized Persulfides

Persulfides react with oxidants, in particular H_2_O_2_, to form perthiosulfenic (RSSOH), perthiosulfinic (RSSO_2_H), and perthiosulfonic (RSSO_3_H) acids (Figure 2). Such oxidative forms have been already identified in several proteins such as MST, papain, albumin, and glutathione peroxidase [21,22,64,65]. The formation of cysteine perthiosulfenic acid (CysSSOH) upon oxidation of protein persulfides has been highlighted in vivo using dimedone alkylation [66]. The authors have compared the levels of dimedone adducts, measured by Western blot using an α-dimedone antibody, in samples reduced or not, knowing that CysS-dimedone adducts cannot be reduced unlike CysSS-dimedone adducts [66]. They also confirmed the presence of CysSSH in some protein tyrosine kinases and their oxidation to CysSSOH in response to the activation of NADPH oxidase [66]. Finally, they proposed that the oxidation of protein cysteine persulfides may represent a novel redox PTM. In another study, a significant elevation of the cysteine perthiosulfonic acid (CysSSO_3_H) levels was measured in the liver *TrxR*/*GR*-null mice compared to WT and *GR*-null mice [55]. This indicated that such oxidized cysteine persulfides are reducible and that reduction mainly relies on the TRX system, at least in this organism [55]. This is in accordance with in vitro data showing that the different forms of oxidized persulfides are reduced to thiols by common reductants and more particularly by TRXs [24,48]. Altogether, these observations suggest that the formation of persulfides may represent a protective mechanism of protein thiols against irreversible oxidation [4]. This might be different from the protective effect described for instance for glutathionylation or other redox PTMs, since such a process has the advantage of also removing more oxidant molecules. The high number of persulfidated proteins identified in plants may thus represent a significant detoxification/ROS buffering mechanism.

### 3.3. TRXs and GRXs as Players of Persulfidation Through H_2_S Synthesis and/or Transpersulfidation Reactions

As already mentioned, protein depersulfidation by TRXs releases H_2_S, which may serve the non-enzymatic persulfidation of oxidized proteins. While this may appear as a futile cycle, this may be a convenient way to maintain stable levels of H_2_S, which can readily react with oxidized proteins generated, for instance, during an oxidative burst. Another pathway, in which TRXs might be connected to the H_2_S-mediated protein persulfidation, is via their interactions with STRs such as MSTs. In addition to the persulfidase function proposed for the human MST isoform [46], MST orthologs from various organisms have been linked to H_2_S synthesis via their interaction with reducing systems [48,67,68]. MSTs are particular STRs composed of two Rhd domains, with only the C-terminal one possessing the conserved catalytic cysteine [1]. MSTs catalyze the conversion of 3-mercaptopyruvate (3-MP), formed by cysteine aminotransferases (CATs) from L-cysteine or by D-amino acid oxidases (DAOs) from D-cysteine, into pyruvate [68,69]. In addition to enzymes of the transsulfuration pathway, cystathionine β-synthase, and cystathionine γ-lyase, the MST/CAT pathway is considered the third pathway of H_2_S synthesis in mammals [68]. On the contrary, the DAO pathway may only be restricted to some specific cells/tissues [69]. However, both reactions lead to the persulfidation of the catalytic cysteine, and then to the production of H_2_S upon subsequent interaction with TRXs [70,71,72,73]. Indeed, the majority of TRXs employ two catalytic cysteines to reduce disulfides. Hence, the persulfide group formed on the catalytic cysteine of TRXs after the first step should be short-lived because of the presence of a resolving cysteine and thus should lead to the release of H_2_S.

In plants, a similar connection between MST isoforms (STR1 and STR2) and the TRX system seems true. Indeed, mitochondrial STR1 has been repeatedly isolated by pull-down approaches as a partner of TRX including the mitochondrial TRXo1 isoform from Arabidopsis [74]. Moreover, the STR1-TRXo1 interaction in mitochondria and the STR2-TRXh1 interaction in the cytosol have been confirmed in vivo using bimolecular fluorescence complementation experiments [75]. The determination of the kinetic parameters for H_2_S production by the plant and human MST/TRX couples revealed an efficient reaction and an affinity between both proteins in a physiological micromolar range [48,72,73]. Moreover, similar kinetic parameters were determined for Arabidopsis MST isoforms when using a GSH/GRX reducing system [48], while the reaction is two orders of magnitude lower with reduced glutathione (GSH) alone, i.e., without GRX [48]. GSH is inoperative with mammalian MSTs [73]. In fact, several STRs possessing a single Rhd domain, such as human TSTD1 and Arabidopsis STR16 and STR18, have been reported to interact with TRXs [45,75]. All these data support the role of TRXs in the H_2_S-dependent protein persulfidation by allowing H_2_S release from MSTs and possibly from other STRs. A potential role of the GSH/GRX system in these reactions remains hypothetical since only in vitro evidence exist so far. Still, despite the absence of evidence for an interaction in vivo between MSTs and GRXs, natural fusion proteins containing both protein domains exist in some prokaryotes. If the involvement of GRXs was confirmed, this may have a significant implication on the nature of the sulfur compound produced by MSTs. Indeed, unlike the majority of TRXs that employ two catalytic cysteines, most GRXs, even those with two cysteines in their characteristic signature, employ a single cysteine for catalysis. Hence, a persulfide group formed on the catalytic cysteine of a GRX is more readily prone to react with GSH forming GSSH or with other proteins, possibly catalyzing transpersulfidation reactions. This may be convenient to achieve an additional degree of specificity in the mechanisms of protein persulfidation because a contribution of TRXs would rather be indirect through H_2_S synthesis and subsequent reaction with oxidized thiol groups while a contribution of GRXs would rather be direct via transpersulfidation.

## 4. Conclusions

Persulfidation represents an evolutionarily conserved redox modification of protein cysteine residues. Numerous studies have deciphered and underlined the diversity and the complexity of molecular mechanisms involved. The progress in our knowledge has also raised new questions. First of all, the relative importance of each identified pathway leading to protein persulfidation is yet largely unknown. H_2_S might be considered as a non-specific agent promoting general protein persulfidation while enzymes catalyzing transpersulfidation reactions might be involved in more specific and controlled pathways of protein persulfidation. The co-translational persulfidation catalyzed by CARS proteins would be complementary to H_2_S-signaling and transpersulfidation-related mechanisms. Indeed, under oxidative conditions, the synthesis of proteins under persulfidated rather than reduced state, would directly protect them from irreversible oxidation. Moreover, many proteins have been identified as persulfidated in several model organisms grown in normal or stress conditions. Nevertheless, for a large majority of these proteins, the identity of persulfidated cysteine residues is still unknown. Considering H_2_S as a mediator converting some redox PTMs into persulfidated proteins, it seems important to evaluate their relationships and, in particular, both the stoichiometry and dynamics of these multiple redox PTMs under diverse physiological conditions and at different time scales. The physiological function of persulfidation is also linked to its transient nature. To date, TRXs seem to be at the same time mediators of protein depersulfidation and of persulfidation, at least through their role in H_2_S synthesis. Some TRXs, notably TRP14, have been suggested to act as specific depersulfidases. Nevertheless, considering the number and diversity of persulfidated proteins in different organisms, there is no doubt that other TRXs or reductases are involved in depersulfidation. Further studies are thus necessary to investigate the nature and specificity of reductases in depersulfidation. This is particularly true in plants that display expanded GRX and TRX families.
